# Bedbound Status During the Last Year of Life Among Community-Dwelling Older Adults

**DOI:** 10.1001/jamanetworkopen.2025.49063

**Published:** 2025-12-19

**Authors:** Katherine A. Ornstein, Mary Louise Pomeroy, Hanna Charankevich, Po-Jen Kung, Orla C. Sheehan, Christine S. Ritchie, Bruce Leff, Jennifer M. Reckrey

**Affiliations:** 1 Center for Equity in Aging, School of Nursing, Johns Hopkins University, Baltimore, Maryland; 2 Department of Medicine for the Older Person, Royal College of Surgeons in Ireland (RCSI) and Connolly Hospital, Dublin, Ireland; 3 Division of Geriatric Medicine and Gerontology, Department of Medicine, Johns Hopkins University School of Medicine, Baltimore, Maryland; 4 Mongan Institute Center for Aging and Serious Illness, Massachusetts General Hospital, Boston; 5 Division of Geriatric Medicine and Palliative Care, Department of Medicine, NYU Grossman School of Medicine, New York, New York

## Abstract

**Importance:**

As older adults live longer in the community, many, particularly those with dementia, may become bedbound at the end of life and require extensive support from caregivers.

**Objective:**

To estimate the prevalence and associated characteristics of bedbound status during the last year of life among community-dwelling decedents, and to examine differences by dementia status.

**Design, Setting, and Participants:**

This cross-sectional study used data from rounds 1 through 13 from the National Health and Aging Trends Study to identify individuals aged 65 years or older who were community-dwelling 12 months before death between the years 2011 and 2023. Data were analyzed between December 2024 and August 2025.

**Main Outcomes and Measures:**

The primary outcome was bedbound status, defined by self or proxy report incorporating frequency, help required, and level of difficulty leaving bed and/or bedroom. Probable dementia was defined using an algorithm that includes cognitive testing and diagnosis history. The number of months between each participant’s date of death and their last interview prior to death was calculated. Bedbound prevalence was estimated by dementia status in monthly intervals up to 1 year before death, along with the amount of caregiver help received. Survey-weighted multivariable regression was used to estimate the likelihood of being bedbound during the last year of life by sociodemographic, clinical, and health characteristics.

**Results:**

Among 3168 decedents (mean [SD] age, 83.0 [0.2] years, 51.9% female; 36.1% [95% CI, 34.7%-39.0%] with dementia), 16.6% (95% CI, 15.0%-18.3%) were bedbound during their last year of life, representing nearly 2.6 million decedents. As individuals with dementia neared death, bedbound prevalence increased from 28.6% (95% CI, 13.9%-47.4%) in the 12 months prior to death to 77% (95% CI, 56.4%-91.6%) in the last month of life. Probable dementia was associated with nearly 5 times greater odds of being bedbound in the last year of life compared with having possible or no dementia (odds ratio, 4.58; 95% CI, 3.09-6.79; *P* < .001). Bedbound decedents received nearly 3 times as many hours of care per week compared with individuals who were not bedbound (98.00 [95% CI, 88.86-107.14] vs 34.03 [95% CI, 31.74-36.32] h/wk; *P* < .001).

**Conclusions and Relevance:**

In this cross-sectional study of decedents, being bedbound during the last year of life was common among individuals with dementia living in the community and imposed substantial demand on caregivers. These findings underscore the urgent need for policies that ensure access to comprehensive home-based care and services to support patients and families.

## Key Points

**Question:**

What is the prevalence of and what are the characteristics associated with being bedbound during the last year of life among a national sample of community-dwelling older adults?

**Findings:**

In this cross-sectional study of 3168 decedents, individuals with dementia had nearly 5 times higher odds of being bedbound compared with those without dementia, with prevalence rising from 21% to 77% from 12 to 0 months before death. Bedbound decedents received nearly 3 times more weekly caregiving hours than those who were not bedbound.

**Meaning:**

These findings suggest that being bedbound during the last year of life is common among older adults with dementia and highlights the urgent need for expanded home-based care and caregiver support services.

## Teaser

This cross-sectional study assesses the characteristics associated with bedbound status during the last year of life among community-dwelling older adults.

## Introduction

Older adults are increasingly living and dying in the community with complex illnesses, including dementia.^1,2,3,4^ Aligned with a national shift away from residential care settings, there are now more homebound older adults than there are older adults living in nursing homes, including many living with dementia (30%-50%).^5^ As disease advances and there is greater functional decline, some community-dwelling older adults become not only homebound, but also bedbound (ie, confined to bed for an extended period).^6^ Being bedbound is associated with frequent complications,^7,8,9,10^ including poorer quality of life,^11^ higher costs of care,^12^ and reduced life expectancy.^12,13^ Older adults who are bedbound may require multiple caregivers for personal care, raising concerns about caregiver strain,^14,15^ emotional stress,^16^ physical injuries,^17^ exhaustion,^18^ poor mental health,^18,19^ social isolation,^20^ financial strain,^9,14^ and caregivers’ ability to tend to their own physical health needs.^15^

It is uncertain how many community-dwelling older adults are bedbound in the US. This is due in part to a lack of clear terminology (eg, bedbound,^12,19^ bedridden,^6,15,21^ bedfast,^22^ bed rest,^23,24^ prolonged immobilization,^25^ or taking to bed^23,26^) and conceptual ambiguity.^16,27,28,29^ Despite a growing number of US individuals dying at home^30^ and increasing investment in Medicaid-funded home and community-based services,^31,32^ nearly all relevant research on bedbound status focuses on nursing home populations.^16,33^ Persons with dementia, 80% of whom live in the community,^34^ are likely at high risk of becoming bedbound, with an estimated bedbound prevalence of 10% among those with severe dementia.^12^ While research on bedbound older adults tends to focus on guidance for best practice in clinical management (eg, reducing pressure ulcers)^7,8,35,36,37,38^ or relates to specific diagnoses (eg, dementia or cancer),^11,19,39,40^ there is scant information on bedbound patients in the general US population, even at the end of life.^23^

The goal of this study was to characterize bedbound status and its prevalence during the last year of life among a general population of community-dwelling decedents. Measures of bedbound status have typically relied on clinical assessments of function and performance—often in disease-specific settings^23,41,42,43,44^—and may, therefore, be less suitable for population-level surveillance and planning for community-dwelling older adults. To more accurately estimate the population of community-dwelling, bedbound decedents across the US and describe their care needs, we developed a measure of bedbound status that considers the frequency of leaving one’s bed and/or bedroom, the amount of help required, and the level of difficulty involved. We hypothesized that dementia status would be associated with being bedbound during the last year of life and that bedbound individuals would require more caregiver support than other community-dwelling decedents.

## Methods

### Design

This cross-sectional study used longitudinal data from the National Health and Aging Trends Study (NHATS), an annual nationally representative survey of Medicare beneficiaries aged 65 years and older. We identified decedents using assessments of last-month-of-life (LML) care completed by proxy respondents. For each deceased person, the month and year of death were reported by a proxy. We considered the last day of the month of death (eg, January 31) as the death date. All NHATS participants provided written informed consent. The study was reviewed and exempted by the Johns Hopkins University School of Medicine institutional review board and followed the Strengthening the Reporting of Observational Studies in Epidemiology (STROBE) reporting guideline.

Our sample included 3459 individuals ages 65 years and older who died between 2012 and 2023 (the most recent wave of data available) and who were community-dwelling (ie, not living in a nursing home) at the interview before their LML assessment.^45^ Given our interest in bedbound status in the last 12 months of life, we excluded 2 decedents with incomplete data on mobility and dementia status and 289 decedents who did not have an interview completed within 12 months before death. Participant characteristics were drawn from the last interview before death (mean [SD], 6.23 [0.07] months before death).

### Measures

#### Assessment of Bedbound Status

**Figure 1.**
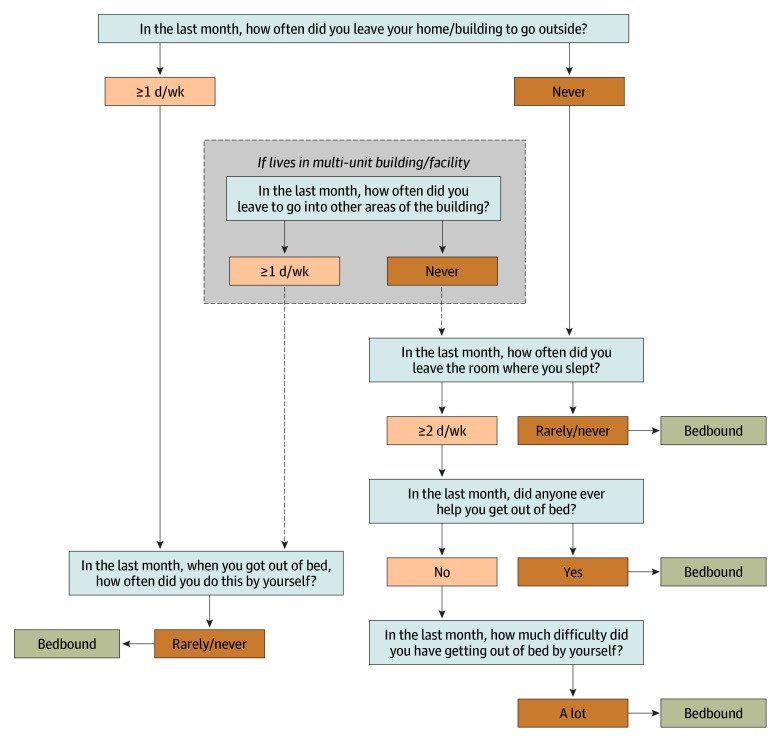
Assessment of Bedbound Status Using National Health and Aging Trends Study Core Interview Flowchart shows the National Health and Aging Trends Study variables used to classify bedbound status. The survey flow and skip logic direct respondents to specific questions about bedbound status on the basis of their answers to prior questions regarding homebound status.

Recognizing the clinical, caregiving, contextual, and lifespace factors that shape bedbound status,^46^ we developed a multifactorial conceptualization of bedbound status that considers frequency, assistance needed, and level of difficulty leaving the bed during the last month. First, in line with previous NHATS research on bedbound status,^12^ we classified individuals who never or rarely left the room where they slept as bedbound. Drawing on our team’s clinical expertise and our interest in the implications of bedbound status on caregiver support, we further classified individuals as bedbound if they never left home and experienced what they indicated as a lot of difficulty getting out of bed; never left home and required help to get out of bed; or if they reported never or rarely got out of bed on their own, even if they did leave home. Figure 1 displays the NHATS survey flow and specific variables included in the last interview before death that were used to classify bedbound status.

#### Dementia Status

Dementia was identified using a validated NHATS classification algorithm.^47^ Respondents were classified as having no dementia, possible dementia, or probable dementia on the basis of the following criteria: (1) a self-report of a physician’s diagnosis of dementia or Alzheimer disease; (2) a proxy-rated score on the Ascertain Dementia 8 screening interview assessing memory, temporal orientation, judgment, and function^48,49^; and (3) scores from cognitive tests evaluating memory (immediate and delayed 10-word recall), orientation (eg, date, month, year, and day of the week), and executive function (clock-drawing test).^47^ Respondents classified as having probable dementia were included in the dementia subgroup in our analyses.

#### Help Received

When NHATS participants or their proxies reported needing help with basic activities of daily living (ADLs) or instrumental ADLs (IADLs) in the past month, the helper was identified. Information about each helper was then obtained, including relationship to the participant, hours of help provided, and whether care was paid (either by insurers like Medicaid or self-funded) or unpaid. We calculated average weekly hours of help received per month by all helpers, the total number of helpers engaged in care, and whether the decedent received any paid help.

#### Participant Characteristics

We examined the following sociodemographic characteristics: age at death, sex, marital status (married or partnered vs other), and a joint measure of race and ethnicity (Hispanic, non-Hispanic Black, non-Hispanic White, or non-Hispanic other race). The other race category encompassed American Indian, Alaska Native, Asian, Native Hawaiian, Pacific Islander, and additional races, although no further breakdown was available. We also included education level (less than a high school degree or General Educational Development degree vs higher), an indicator of whether total income of an individual or household fell within the lowest income quartile of income distribution in our sample, living in a metropolitan area, self-reported enrollment in Medicaid, and whether the individual lived alone in a private residence. We examined residential settings by classifying residences as residential settings (eg, group homes, assisted living facilities, and continuing care retirement communities) or private homes according to interviewer observations and questions of the participant or facility staff.

We also examined various clinical and health characteristics. Individuals were dichotomized as frail vs robust and/or prefrail using the physical frailty phenotype.^50,51^ Presence of depressive symptoms and anxiety were assessed using the Patient Health Questionnaire–2 and the Generalized Anxiety Disorder 2-item screening instruments, respectively.^52^ Functional status was assessed using count variables that summed the number of ADLs and the number of IADLs with which the participant received help and/or had difficulty. Pain was assessed on the basis of whether the individual had been bothered by pain in the last month and whether pain ever limited their activities. A count of 9 self-reported chronic conditions (heart attack, heart disease, high blood pressure, arthritis, osteoporosis, diabetes, lung disease, stroke, and cancer) was dichotomized into a variable that classified individuals as having 0 to 1 vs 2 or more chronic conditions other than dementia. An indicator of dual sensory impairment was also examined according to whether an individual was able to read newspaper print (visual impairment) and carry on a conversation while a TV or radio was on while using appropriate visual or hearing aids (hearing impairment). We also included a continuous measure of body mass index according to reported weight and height (calculated as weight in kilograms divided by height in meters squared), as well as an indicator of self-rated health (poor or fair vs good, very good, or excellent).

### Statistical Analysis

All empirical analyses were performed using RStudio statistical software version 4.5.0 (R Project for Statistical Computing). Analyses were conducted between December 2024 to August 2025. All analyses were survey-weighted to adjust for unequal probabilities of participant selection and to account for the complex survey design variables. We calculated the number of months between each participant’s date of death and the date of their last interview before death and grouped participants according to how many months had passed between their last interview and death. Using this information, we estimated the prevalence of bedbound status among the population of decedents—both overall and by dementia status—at monthly intervals in the year prior to death. We also examined the amount of help received by respondents by bedbound status by each of the last 12 months of life, reporting estimates with 95% CIs. Notably, because NHATS conducts annual assessments, bedbound status could only be ascertained during 1 month in the last year of life per respondent (at the last survey interview before death).

To conduct statistical comparisons by bedbound status, we aggregated individuals who were classified as bedbound in any month in the year prior to death and compared them with those who were not classified as bedbound prior to death. Characteristics of individuals who were and were not bedbound in the last year of life were compared using *t* tests. Similarly, we compared help received by bedbound status per month and estimated linear trends in hours of help received over the last year of life by month. We used multivariable logistic regression to evaluate how the likelihood of being bedbound in the last year of life was associated with sociodemographic, clinical, and health characteristics and months until death. Using survey weighted logistic regression we then calculated the mean estimated probability of being bedbound by dementia status in the last year of life conditional on sociodemographic, clinical, and health characteristics (eFigure 2 in Supplement 1). Measures with low variation (ie, ADL and IADL dependency) were not included in regression analyses. Statistical significance was set at 2-sided *P* < .05 for all analyses.

## Results

Among a sample of 3168 decedents, 1644 were female (51.9%; 95% CI, 49.6%-54.2%) and 1524 were male (48.1%; 95% CI, 45.8%-50.4%), 1143 (36.1%; 95% CI, 34.7%-39.0%) had dementia, and the mean (SD) age at death was 83.0 (0.2) years. We identified 590 community-dwelling decedents (18.6%, unweighted) who were bedbound for at least 1 month during their last year of life. This included 362 individuals who reported they never or rarely got out of bed by themselves, 106 who never or rarely left the room where they slept, 115 who never left their home and required help to get out of bed, and 7 who never left their home and had a lot of difficulty getting out of bed by themselves. Applying NHATS survey weights, this represents 2 518 076 million community-dwelling individuals who were bedbound at least 1 month during their last year of life, or 16.6% (95% CI, 15.0%-18.3%) of all community-dwelling decedents aged 65 years or older in the US.

The characteristics of decedents stratified by bedbound status are included in Table 1. Decedents who were bedbound during the last year of life were different from decedents who were nonbedbound across several sociodemographic, clinical, and health characteristics. For example, bedbound individuals were more likely to be female (bedbound: 63.0%; 95% CI, 57.5%-68.1%; not bedbound: 49.8%; 95% CI, 47.2%-52.3%), non-Hispanic Black (bedbound: 11.1%; 95% CI, 9.2%-13.5%; not bedbound 8.8%; 95% CI, 7.9%-9.7%), and have Medicaid (bedbound: 31.9%; 95% CI, 27.1%-37.1%; not bedbound: 18.4%; 95% CI, 16.4%-20.6%). Those who were bedbound were more likely to be frail (bedbound: 77.9%; 95% CI, 72.9%-82.1%; not bedbound: 40.7%; 95% CI, 38.2%-43.2%) and have dementia (bedbound: 78.9%; 95% CI, 74.0%-83.1%; not bedbound: 28.5%; 95% CI, 26.3%-30.7%).

**Table 1.** Sociodemographic, Clinical, and Health Characteristics of Decedents by Their Bedbound Status in the Last Year of Life^a^

Characteristic	Bedbound (n = 590)		Not bedbound (n = 2578)		Difference, %	*P* value
	Observed No. (weighted %)	Missing count^b^	Observed No. (weighted %)	Missing count		
Sociodemographic characteristics						
Time from last interview to death, mean (SD), mo	4.9 (0.2)	0	6.5 (0.1)	0	1.64	<.001
Age at death, mean (SD), y	85.4 (0.5)	0	82.5 (0.2)	0	2.93	<.001
Gender						
Female	389 (63.0)	0	1339 (49.8)	0	13.23	<.001
Male	201 (37.0)	0	1239 (50.2)	0	13.23	
Race and ethnicity						
Black (non-Hispanic)	156 (11.1)	0	525 (8.8)	0	2.36	.05
Hispanic	56 (11.9)	0	121 (5.6)	0	6.22	<.001
Other (non-Hispanic)^c^	11 (4.7)	0	69 (3.6)	0	1.15	.53
White (non-Hispanic)	353 (69.5)	0	1823 (78.9)	0	−9.31	<.001
Married^d^	164 (31.7)	0	943 (41.9)	0	−10.17	<.001
Less than high school education	360 (60.6)	17	1494 (55.9)	38	4.74	.12
Income in lowest quantile	236 (36.7)	0	788 (27.1)	0	9.65	<.001
Lives in metropolitan area	404 (80.3)	82	1778 (81.1)	372	−0.76	.78
Lives alone in private residence^d^^,^^e^	70 (14.2)	0	818 (30.8)	0	−16.64	<.001
Medicaid^d^	195 (31.9)	35	480 (18.4)	163	13.49	<.001
Clinical and health characteristics^d^						
Probable dementia	487 (78.9)	0	836 (28.5)	0	50.43	<.001
Frail	482 (77.9)	0	1097 (40.7)	0	37.20	<.001
Body mass index, mean (SD)^f^	21.3 (0.6)	0	25.2 (0.3)	0	−3.9	<.001
Depressive symptoms^g^	258 (47.5)	40	626 (24.5)	48	23.09	<.001
Anxiety^g^	187 (35.6)	48	460 (18.2)	46	17.40	<.001
No. of chronic conditions, mean (SD)^h^	3.5 (0.1)	0	3.1 (0.04)	0	0.36	<.001
Activities of daily living score, mean (SD)^i^	5.4 (0.04)	0	2.5 (0.05)	0	2.90	<.001
Instrumental activities of daily living score, mean (SD)^g^	3.7 (0.08)	0	1.5 (0.04)	0	2.23	<.001
Pain limits activity	379 (64.4)	5	1496 (59.5)	8	4.86	.08
Dual hearing and/or vison impairment	86 (12.9)	0	95 (3.5)	0	9.44	<.001
Self-rated poor and/or fair health	220 (41.8)	1	422 (17.7)	6	24.13	<.001

^a^ The total sample is constructed using National Health and Aging Trends Study rounds 2012 to 2023 and includes 2011, 2015 and 2022 cohorts.

^b^ Missing responses indicate inapplicable, missing, “don’t know,” or refused to answer.

^c^ The category other (non-Hispanic) includes American Indian, Alaska Native, Asian, Native Hawaiian, Pacific Islander, and additional groupings with no further breakdown available as this variable was obtained directly from the National Health and Aging Trends Study.

^d^ Variable derived from the interview completed prior to the last month of life interview.

^e^ Captures individuals who were living alone in the community, not in assisted living or other congregate residential care facilities, in the year before death.

^f^ Body mass index is calculated as weight in kilograms divided by height in meters squared.

^g^ Symptoms of depression and anxiety were measured using the Patient Health Questionnaire and Generalized Anxiety Disorder 2-item screening instruments, respectively.

^h^ Chronic conditions include heart attack, heart disease, high blood pressure, arthritis, osteoporosis, diabetes, lung disease, stroke, and cancer.

^i^ Activities of daily living include help or difficulty with eating, bathing, toileting, dressing, and getting out of bed. Instrumental activities of daily living include doing laundry, preparing meals, shopping, handling bills and banking, and taking medications by oneself with or without difficulty.

**Figure 2.**
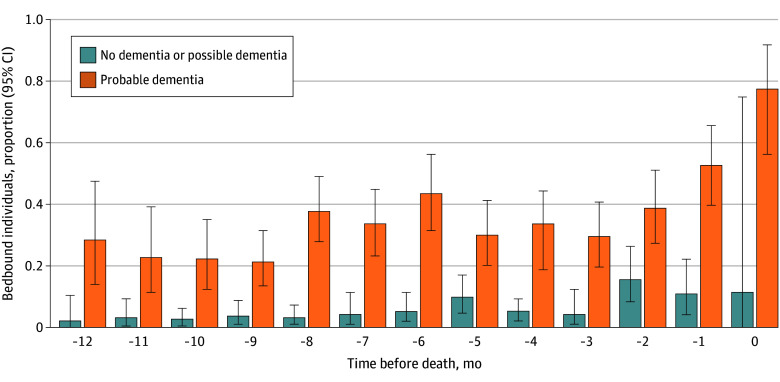
Proportion of Bedbound Older Adults in the Months Before Death, by Dementia Status Error bars denote 95% CIs.

The monthly prevalence of bedbound status during the last year of life by dementia status can be found in Figure 2. As individuals got closer to death, bedbound prevalence increased from approximately 28.6% (95% CI, 13.9%-47.4%) at 12 months prior to death to 77.4% (95% CI, 56.4%-91.6%) in the last month of life among those with dementia and from 1.9% (95% CI, 0.1%-10.2%) to 11.5% (95% CI, 0.002%-74.1%) among those without dementia. Individuals with dementia were far more likely to be bedbound than those without dementia at any month during the last year of life (eg, 6 months before death 43.5% vs 5.3%; difference in means 36.9% (95% CI, 25.8%-50.7%; *t *= 6.05; *P *< .001) (eTable in Supplement 1). 

Bedbound decedents received more hours of care compared with nonbedbound decedents throughout the last year of life (eFigure 1 in Supplement 1). Decedents who were bedbound during the year prior to death received care from a mean of 3.02 helpers (95% CI, 2.86-3.18 helpers) vs 2.18 helpers (95% CI, 2.10-2.25 helpers) among those who were not bedbound (*P* < .001). Similarly, bedbound decedents received 3 times as many total hours of care per week (98.00 [95% CI, 88.86-107.14] h/wk vs 34.03 [95% CI, 31.74-36.32] h/wk; *P* < .001). Bedbound decedents also were more likely to receive paid care (45.15% [95% CI, 39.9%-50.4%] vs 18.31% [95% CI, 16.3%-20.3%]; *P *<.001) and received a higher number of hours of paid care (20.92 [95% CI, 16.4-25.5] h/wk vs 3.71 [95% CI, 2.8-4.6] h/wk; *P* < .001) than nonbedbound individuals (Table 2).

**Table 2.** Characteristics of Help Received Among Decedents in Their Last Year of Life, by Bedbound Status^a^

Variable	Mean (95% CI)			*P* value
	All (N = 3168)	Bedbound (n = 590)	Nonbedbound (n = 2578)	
Any paid help, % of decedents	23.03 (21.08-24.97)	45.15 (39.94-50.36)	18.31 (16.31-20.31)	<.001
Total help, h/wk	45.27 (42.58-47.96)	98.00 (88.86-107.14)	34.03 (31.74-36.32)	<.001
Total paid help, h/wk	6.73 (5.63-7.84)	20.92 (16.35-25.49)	3.71 (2.83-4.59)	<.001
No. of helpers	2.32 (2.25-2.39)	3.02 (2.86-3.18)	2.18 (2.10-2.25)	<.001
No. of paid helpers	0.31 (0.28-0.34)	0.61 (0.52-0.69)	0.24 (0.21-0.27)	<.001

^a^ All percentages and means are survey weighted.

**Figure 3.**
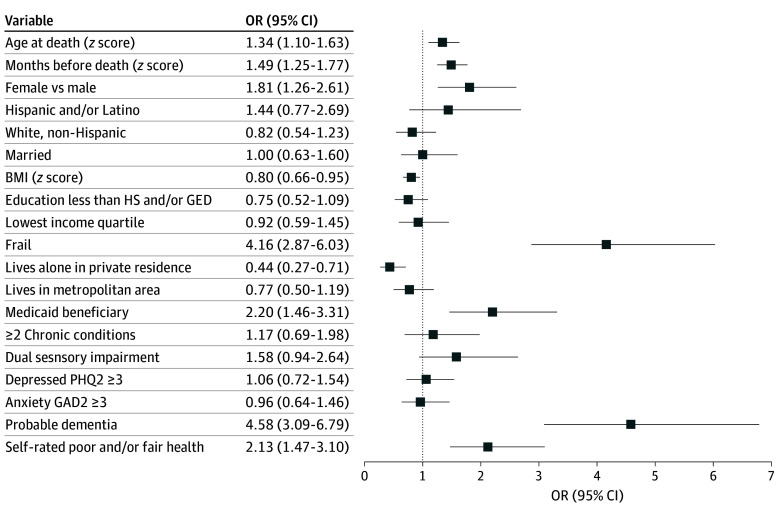
Forest Plot Depicting Estimated Odds Ratios (ORs) of Bedbound Status in the Year Before Death Using Survey-Weighted Multivariable Logistic Regression BMI indicates body mass index; GAD-2, Generalized Anxiety Disorder 2-item; GED, General Educational Development; HS, high school; PHQ2, Patient Health Questionnaire–2.

The overall estimated probability of bedbound status at any month in the year prior to death among those with dementia was 23.5% (eFigure 2 in Supplement 1). In adjusted multivariable regression analyses, compared with individuals with possible or no dementia, individuals with probable dementia had nearly 5 times increased odds of being bedbound in the last year of life (odds ratio [OR], 4.58; 95% CI, 3.09-6.79; *P* < .001). Other characteristics associated with being bedbound included frailty status (OR, 4.16; 95% CI, 2.87-6.03), female gender (OR, 1.81; 95% CI, 1.26-2.61), being enrolled in Medicaid (OR, 2.20; 95% CI, 1.46-3.31), and self-rated poor or fair health (OR, 2.13; 95% CI, 1.47-3.10). Decedents who lived alone in a private residence were less likely to be bedbound (OR, 0.44; 95% CI, 0.27-0.71) (Figure 3).

## Discussion

This cross-sectional study is among the first population-based studies to examine bedbound status in the last year of life among community-dwelling older US individuals. Our estimation that 2.5 million decedents were bedbound and living in the community has important implications for aging in place, caregiving, and home-based care delivery in general.

Consistent with the progressive functional decline inherent in dementia, we found that community-dwelling individuals with dementia were nearly 5 times more likely to be bedbound at any month during the last year of life compared with those without dementia. This elevated risk of immobility is likely multifactorial, with cognitive impairment exacerbating physical frailty, particularly in home-based care settings. Furthermore, caregivers of persons with dementia may lack the resources or training to safely assist individuals with dementia in staying mobile. For example, concerns about falls, confusion, or resistance to care may lead to less frequent attempts to get the person with dementia out of bed.

We also found that those who were bedbound at any month in the year prior to death required nearly 3 times the number of caregiving hours and additional helpers and had far greater use of paid care. This reflects the high levels of support required to live in the community despite severe functional decline and suggests a potential survivor effect, whereby only those with sufficient medical, social, and caregiving resources can remain in the community while bedbound. Similarly, larger social networks, which may be critical for securing the assistance required to remain at home despite being bedbound, may also help explain the association between female gender and bedbound status.^53,54,55^ Furthermore, Medicaid coverage of nonmedical paid care and durable medical equipment may allow bedbound older adults to remain in the community rather than transition to nursing homes.^31,32^ Interestingly, lower education attainment and living alone in the community were associated with lower odds of bedbound status, which may reflect reduced access to resources, information, and caregiving support that are necessary to remain in the community at the end of life. Further research is warranted to elucidate the complex, and likely bidirectional, associations between bedbound status and key social determinants of health including gender. Identifying the enabling factors that allow individuals with profound disability to remain in community settings is essential for designing equitable, end-of-life care strategies.

To meet the needs of those who are bedbound in the last year of life, we need to understand how to best support their caregivers. Caregivers to bedbound participants report a general lack of training that may precipitate common medical complications associated with bedbound status, such as urinary tract infections and pressure ulcers, further increasing caregiver challenges^9,18,19,21,56^ Furthermore, end-of-life care is highly complex and demanding, especially for persons with dementia and their family caregivers.^57,58^ Symptom management may be especially challenging due to myriad needs; not surprisingly, the majority of family caregivers (78%) who assist with symptom management at the end of life report difficulty.^59^ Caregiver training programs have been shown to improve the ability of caregivers to assist older adults with functional mobility and should be widely accessible as a critical component of community-based care, especially for those with dementia. Given the importance of paid care for people with functional impairment living in the community and those at the end of life,^60,61^ supporting family caregivers to effectively collaborate with paid caregivers has the potential to both improve the care of bedbound older adults and reduce strain among family caregivers.^62^

Our findings reflect the need for in-home support to older adults who are bedbound. While home-based medical care may be uniquely suited to support the complex health needs of bedbound older adults, only 11% of older adults who report rarely or never leaving their homes received any home-based medical care.^63^ Intermittent skilled home health care is far more ubiquitous, especially among those with dementia,^64,65^ and may be filling critical care needs for older adults who wish to remain in the community at the end of life.^66^ Home hospice is another essential care model to control symptoms and provide essential support at the end of life to both older adults and their families.^67^ In addition to these existing care models at home, our findings may be especially important relative to the development and implementation of new dementia care models (eg, the Guiding an Improved Dementia Experience [GUIDE] model) and may impact decision-making regarding end-of-life care. To align care with patient preferences, policy efforts must prioritize scalable strategies to support home-based care, address caregiver strain, and ensure adequate workforce training and resources in the community.

### Limitations

Our study has several limitations. Although the mortality follow-back sampling method introduces potential selection bias,^68^ this approach is feasible for studying bedbound status among decedents. We relied on yearly NHATS assessments, and small sample sizes prevented reliable monthly population estimates. Proxy respondents may have introduced recall bias, and we could not determine the cause or duration of bedbound status. Furthermore, while participant health may have impacted interview timing or completion, we adjusted for time between death and the final NHATS interview. Given recent concerns about NHATS mortality estimates,^69^ our overall estimation of the number of decedents who are bedbound may be an undercount. Our broad definition of bedbound included individuals who always require help getting out of bed, regardless of functional status. While there is no criterion standard definition of bedbound status,^16,27,28,29^ and the NHATS was not specifically designed to measure bedbound status, its detailed mobility and function assessments provide a valuable foundation for estimating bedbound prevalence in the aging US population that can inform dementia and end-of-life care policy. Future research should refine and compare with functional assessments (eg, the Outcome and Assessment Information Set). Additionally, we could only examine bedbound status at 1 point during the last year of life; other time frames and intervals warrant exploration, particularly for those with dementia. For example, bedbound status was not assessed at the last month of life assessment. Furthermore, the chronic, slowly progressive trajectory of dementia may make bedbound status more readily detectable in annual assessments compared with more acute or rapidly disabling conditions (eg, stroke), where brief episodes of being bedbound may be undercaptured.

## Conclusions

In this cross-sectional study of decedents, being bedbound during the last year of life was common among individuals with dementia living in the community and imposed substantial demand on caregivers. Our findings underscore the urgent need for services and policies that ensure access to comprehensive home-based care and dementia-capable services to support older adults and their caregivers.

## Notes

Supplement 1. eTable. Prevalence of Bedbound Status in the Last Year of Life, by Dementia Status eFigure 1. Total Hours of Help Per Week in the Months Before Death by Bedbound Status eFigure 2. Predicted Survey-Weighted Probabilities of Bedbound Status Among Decedents, by Dementia Status

Supplement 2. Data Sharing Statement
