# Supplementary material for: Bedbound Status During the Last Year of Life Among Community-Dwelling Older Adults
Source: JAMA Netw Open. 2025 Dec 19;8(12):e2549063. doi: 10.1001/jamanetworkopen.2025.49063 (PMC12717622; doi:10.1001/jamanetworkopen.2025.49063)
Supplement: Supplement 1. — eTable. Prevalence of Bedbound Status in the Last Year of Life, by Dementia Status eFigure 1. Total Hours of Help Per Week in the Months Before Death by Bedbound Status eFigure 2. Predicted Survey-Weighted Probabilities of Bedbound Status Among Decedents, by Dementia Status [file jamanetwopen-e2549063-s001.pdf]

Supplemental Online Content

Ornstein KA, Pomeroy ML, Charankevich H, et al. Epidemiology of bedbound status during the last year of life among community-dwelling older adults. *JAMA Netw Open.* 2025;8(12):e2549063. doi:10.1001/jamanetworkopen.2025.49063

eTable Prevalence of Bedbound Status in the Last Year of Life, by Dementia Status

eFigure 1. Total Hours of Help Per Week in the Months Before Death by Bedbound Status

eFigure 2. Predicted Survey-Weighted Probabilities of Bedbound Status Among Decedents, by Dementia Status

This supplemental material has been provided by the authors to give readers additional information about their work.

© 2025 Ornstein KA et al. *JAMA Network Open*.

eTable Prevalence of Bedbound Status in the Last Year of Life, by Dementia Status

Number of months to death	Monthly prevalence of bedbound status among persons with dementia[a]				Monthly prevalence of bedbound status among persons with no/possible dementia[b]			
	Frequency	Percent	LL CI	UL CI	Frequency	Percent	LL CI	UL CI
-12	16/59	29%	13.93%	47.46%	1/111	2%	0.05%	10.18%
-11	15/67	23%	11.09%	38.94%	4/149	3%	0.67%	9.35%
-10	20/83	22%	12.15%	35.07%	6/161	2%	0.65%	6.42%
-9	30/117	21%	13.20%	31.30%	5/188	3%	0.81%	8.95%
-8	49/126	38%	27.62%	49.01%	8/179	3%	0.96%	7.40%
-7	45/124	33%	22.99%	44.93%	7/180	4%	0.79%	11.39%
-6	45/122	43%	31.50%	56.01%	8/173	5%	1.90%	11.26%
-5	43/116	30%	20.34%	40.99%	16/160	9%	4.25%	16.02%
-4	53/134	33%	23.57%	44.52%	12/168	5%	2.27%	9.42%
-3	38/105	29%	19.60%	40.94%	6/124	4%	0.80%	12.21%
-2	50/119	39%	27.21%	50.97%	15/126	16%	8.25%	26.09%
-1	54/108	54%	40.33%	66.36%	12/99	11%	4.12%	22.07%
0	30/43	77%	56.42%	91.55%	2/23	11%	0.002%	74.51%

Notes. Estimates reflect raw counts and weighted percentages of bedbound in the interview before the last month of life (LML) interview, by dementia status in the year prior to the LML assessment. Difference in mean bedbound status by dementia status at 6 months prior to death is significant at $p<0.001$.

[a] "Dementia" captures probable dementia cases only. Total number of persons with dementia n=1,323. Total number of bedbound persons with dementia n=488.

[b] "No/possible dementia" captures both non-dementia and possible dementia cases. Total number of persons with no/possible dementia n=1,845. Total number of bedbound persons with no/possible dementia n=102.

© 2025 Ornstein KA et al. *JAMA Network Open*.

eFigure 1. Total hours of Help Per Week in the Months Before Death by Bedbound Status

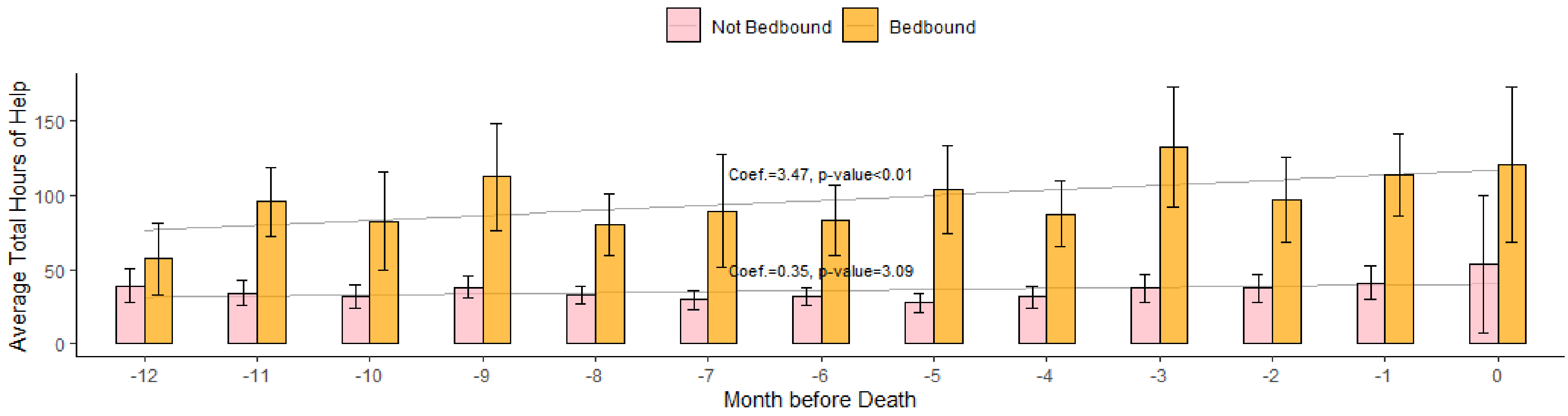

© 2025 Ornstein KA et al. *JAMA Network Open*.

eFigure 2. Predicted Survey-Weighted Probabilities of Bedbound Status Among Decedents, by Dementia Status

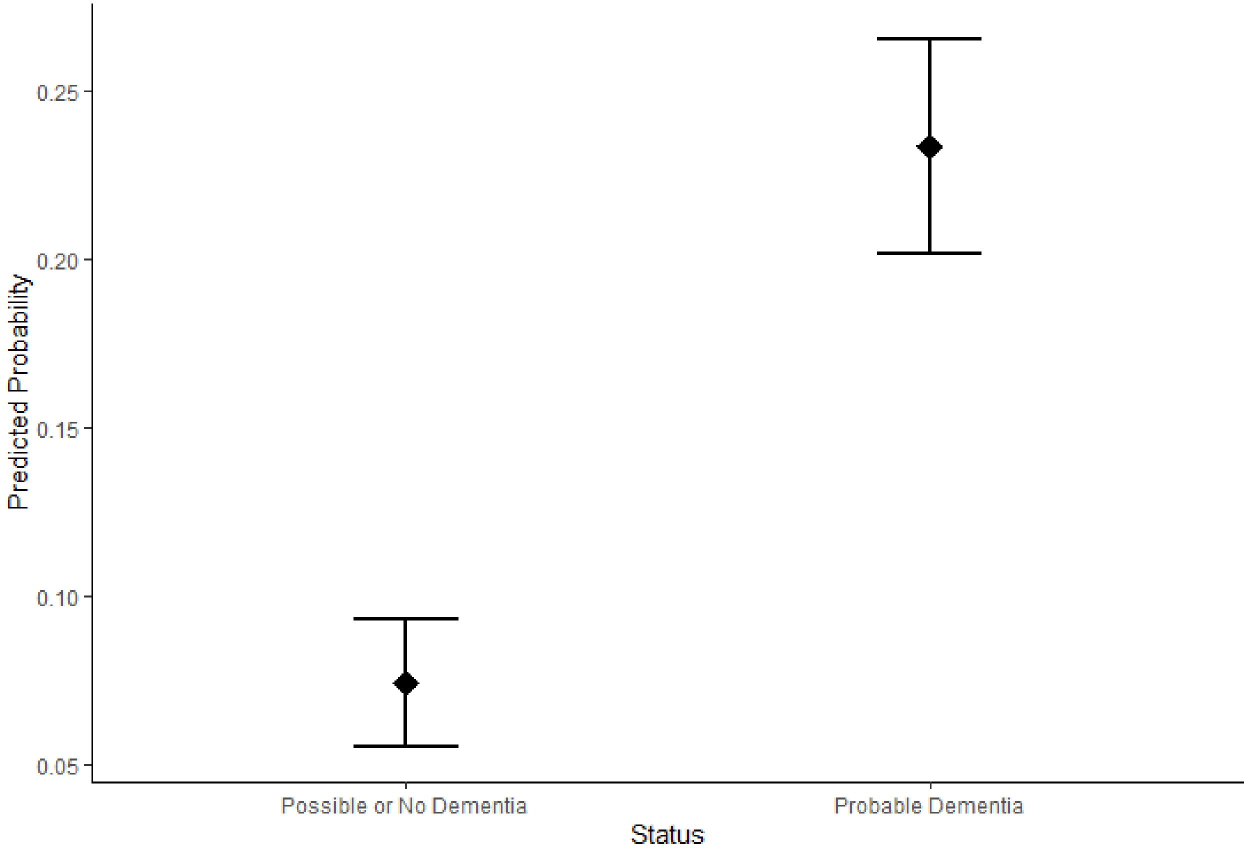

Status	Marginal Mean	SE
Possible or No Dementia	7.42%	0.97%
Probable Dementia	23.36%	1.62%

© 2025 Ornstein KA et al. *JAMA Network Open*.
