# Supplementary material for: Bedbound Status During the Last Year of Life Among Community-Dwelling Older Adults
Source: JAMA Netw Open. 2025 Dec 19;8(12):e2549063. doi: 10.1001/jamanetworkopen.2025.49063 (PMC12717622; doi:10.1001/jamanetworkopen.2025.49063)
Supplement: Supplement 2. — Data Sharing Statement [file jamanetwopen-e2549063-s002.pdf]

Data Sharing Statement

Ornstein. Epidemiology of Bedbound Status During the Last Year of Life Among Community-Dwelling Older Adults. *JAMA Netw Open*. Published December 19, 2025. doi:10.1001/jamanetworkopen.2025.49063

Data
Data available: No

Additional Information
Explanation for why data not available: data are publicly available via NHATS
